# Deep learning‐based segmentation in MRI‐(immuno)histological examination of myelin and axonal damage in normal‐appearing white matter and white matter hyperintensities

**DOI:** 10.1111/bpa.13301

**Published:** 2024-08-23

**Authors:** Gemma Solé‐Guardia, Matthijs Luijten, Esther Janssen, Ruben Visch, Bram Geenen, Benno Küsters, Jurgen A. H. R. Claassen, Geert Litjens, Frank‐Erik de Leeuw, Maximilian Wiesmann, Amanda J. Kiliaan

**Affiliations:** ^1^ Department of Medical Imaging, Anatomy, Research Institute for Medical Innovation, Radboud University Medical Center, Donders Institute for Brain, Cognition & Behavior, Center for Medical Neuroscience, Preclinical Imaging Center PRIME Radboud Alzheimer Center Nijmegen The Netherlands; ^2^ Department of Pathology, Research Institute for Medical Innovation Radboud University Medical Center Nijmegen The Netherlands; ^3^ Department of Geriatrics, Research Institute for Medical Innovation, Radboud University Medical Center, Donders Institute for Brain, Cognition & Behavior Radboud Alzheimer Center Nijmegen The Netherlands; ^4^ Department of Cardiovascular Sciences University of Leicester Leicester UK; ^5^ Computational Pathology Group, Research Institute for Medical Innovation Radboud University Medical Center Nijmegen The Netherlands; ^6^ Department of Neurology, Research Institute for Medical Innovation, Radboud University Medical Center Donders Institute for Brain, Cognition & Behavior, Center for Medical Neuroscience Nijmegen The Netherlands

**Keywords:** deep learning segmentation, myelin microstructure, neurodegeneration, normal‐appearing white matter, small vessel disease, white matter hyperintensities

## Abstract

The major vascular cause of dementia is cerebral small vessel disease (SVD). Its diagnosis relies on imaging hallmarks, such as white matter hyperintensities (WMH). WMH present a heterogenous pathology, including myelin and axonal loss. Yet, these might be only the “tip of the iceberg.” Imaging modalities imply that microstructural alterations underlie still normal‐appearing white matter (NAWM), preceding the conversion to WMH. Unfortunately, direct pathological characterization of these microstructural alterations affecting myelinated axonal fibers in WMH, and especially NAWM, is still missing. Given that there are no treatments to significantly reduce WMH progression, it is important to extend our knowledge on pathological processes that might already be occurring within NAWM. Staining of myelin with Luxol Fast Blue, while valuable, fails to assess subtle alterations in white matter microstructure. Therefore, we aimed to quantify myelin surrounding axonal fibers and axonal‐ and microstructural damage in detail by combining (immuno)histochemistry with polarized light imaging (PLI). To study the extent (of early) microstructural damage from periventricular NAWM to the center of WMH, we refined current analysis techniques by using deep learning to define smaller segments of white matter, capturing increasing fluid‐attenuated inversion recovery signal. Integration of (immuno)histochemistry and PLI with post‐mortem imaging of the brains of individuals with hypertension and normotensive controls enables voxel‐wise assessment of the pathology throughout periventricular WMH and NAWM. Myelin loss, axonal integrity, and white matter microstructural damage are not limited to WMH but already occur within NAWM. Notably, we found that axonal damage is higher in individuals with hypertension, particularly in NAWM. These findings highlight the added value of advanced segmentation techniques to visualize subtle changes occurring already in NAWM preceding WMH. By using quantitative MRI and advanced diffusion MRI, future studies may elucidate these very early mechanisms leading to neurodegeneration, which ultimately contribute to the conversion of NAWM to WMH.

## Graphical abstract

Our study aimed to quantify myelin surrounding axonal fibers and axonal‐ and microstructural damage in detail by combining (immuno)histochemistry with polarized light imaging (PLI). To study the extent (of early) microstructural damage from periventricular NAWM to the center of WMH, we refined current analysis techniques by using deep learning to define smaller segments of white matter, capturing increasing fluid‐attenuated inversion recovery signal. Results showed that myelin loss, axonal integrity, and white matter microstructural damage are not limited to WMH but already occur within NAWM.

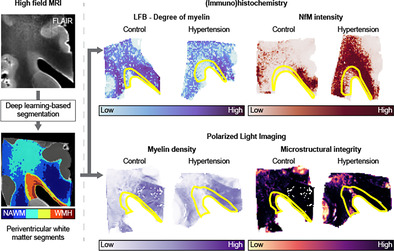

## 1 INTRODUCTION

Cerebral small vessel disease (SVD) causes about a fifth of all strokes and is the major vascular contributor to dementia worldwide [1]. White matter hyperintensities (WMH) of presumed vascular origin are the most common magnetic resonance imaging (MRI) markers of SVD [2, 3]. Their prevalence and burden augment with hypertension, which is the most important risk factor for sporadic SVD [4, 5]. WMH present a heterogenous pathology, including myelin pallor and axonal loss [6, 7]. Previous MRI studies have shown that normal‐appearing white matter (NAWM) is gradually converted into WMH [8, 9]. The MRI findings demonstrate an increased mean diffusivity in these NAWM areas, but they lack specificity of underlying pathophysiological processes. In order to develop effective treatment and prevention strategies, this pathophysiology of WMH needs to be unraveled.

Pathological validation of microstructural alterations such as disruption of myelinated fibers and accumulation of phosphorylated neurofilament, which reflects damaged nerve fibers [10, 11], in NAWM is still missing because early pathological studies have prioritized the assessment of the underlying pathology of WMH (reviewed by [7]), often neglecting NAWM. Traditionally, myelin damage is evaluated with Luxol Fast Blue (LFB) staining [12], but a potential drawback may be that LFB may not be sensitive enough to capture subtle microstructural damage of myelinated axonal fibers [13]. On the other hand, an established technique able to post‐mortem quantify the amount of myelin (density) and fiber microstructural damage is polarized light imaging (PLI) [14, 15, 16, 17].

Similarly, traditional MRI analysis methods capture only the “tip of the WMH iceberg.” WMH are defined by a hyperintense area on a FLAIR image [18], but the distinction between WMH and NAWM may not be that dichotomous, especially in areas where the white matter appears normal on FLAIR. This is in line with observations of gradually lower microstructural integrity, particularly in the border zone between WMH and NAWM [8, 9, 19, 20, 21]. Therefore, the degree of (early) white matter microstructural alterations in NAWM may be better captured by refining image‐histology registration. This could be achieved by careful co‐registration of very small MRI‐histopathological sections in the WMH – NAWM border zone with the aid of deep learning methods [22, 23]. By defining these segments based on increasing FLAIR signal from periventricular NAWM into the center of WMH, it may be possible to capture focal, gradual damage preceding the conversion into WMH.

Therefore, we aimed to investigate the extent of white matter microstructural damage using both (immuno)histochemistry and PLI across periventricular NAWM and WMH, with deep learning‐based segmentation and MRI‐pathological examination in individuals with hypertension and age‐matched controls.

## 2 MATERIALS AND METHODS

### 2.1 Cases

Between 2015 and 2019, 17 individuals with hypertension (according to guidelines at that time [24]) and 5 age‐matched control individuals were included in this study through the body donors' program at the Radboud University Medical Center, Nijmegen, the Netherlands. Table 1 shows the demographic and clinical characteristics of the study cohort. All protocols concerning data acquisition and tissue processing were approved by the Medical Ethics Review Committee (Commissie Mensgebonden Onderzoek (CMO) region Arnhem‐Nijmegen).

**TABLE 1:** Demographic and clinical characteristics of the study cohort.

	Total (*n* = 22)	Controls (*n* = 5)	Individuals with hypertension (*n* = 17)	*p* value
Demographics				
Age, mean ± SD, years	80.6 ± 8.1	80.2 ± 8.6	80.7 ± 8.2	0.905
Sex, female, *n* (%)	10 (45.5%)	3 (60.0%)	7 (41.2%)	0.457
*Post‐mortem* delay, mean ± SD, hours	22.4 ± 6.8	20.0 ± 2.9	23.1 ± 7.4	0.375
Risk factors				
BMI, mean ± SD, kg/m^2^	23.1 ± 4.4	23.8 ± 3.8	22.9 ± 4.7	0.756^a^
Diabetes, *n* (%)	5 (22.7%)	1 (20.0%)	4 (23.5%)	0.869
Hypercholesterolemia, *n* (%)	11 (50.0%)	1 (20.0%)	10 (58.8%)	0.127
Smoking, *n* (%)	7 (31.8%)	2 (40.0%)	5 (29.4%)	0.655
Alcohol use, *n* (%)	3 (13.6%)	0 (0%)	3 (17.6%)	0.312
Imaging				
Fazekas score, moderate to severe WMH (Score ≥2), *n* (%)	10 (45.5%)	0 (0%)	10 (58.8%)	0.020*
WMH volume^b^, mean ± SD, mL	1.40 ± 0.68	0.91 ± 0.26	1.54 ± 0.70	0.020*

Abbreviations: BMI, body mass index; SD, standard deviation; WMH, white matter hyperintensity.

^a^ Data missing for *n* = 6 for BMI.

^b^ These measures correspond to the WMH volume from the left hemisphere.

* *p* < 0.05.

### 2.2 Post‐mortem MRI

Details on tissue processing and post‐mortem MRI data acquisition have been previously described [25]. The left hemisphere was scanned post‐mortem at room temperature on a Bruker 7 Tesla Clinscan MR system (Bruker Biospin, Ettlingen, Germany) interfaced with a Siemens Syngo VB15 console. The protocol included several sequences, including T1‐weighted sequence (repetition time 20 ms, echo time 1 ms, voxel size 400 × 400 × 400 μm isotropic) and T2‐weighted FLAIR sequence (repetition time 8200 ms, echo time 39 ms, 2 averages, voxel size 500 × 500 × 500 μm), which were used in this study.

#### 2.2.1 Deep‐learning segmentation of post‐mortem MRI of periventricular WMH and NAWM into segments

**FIGURE 1:**
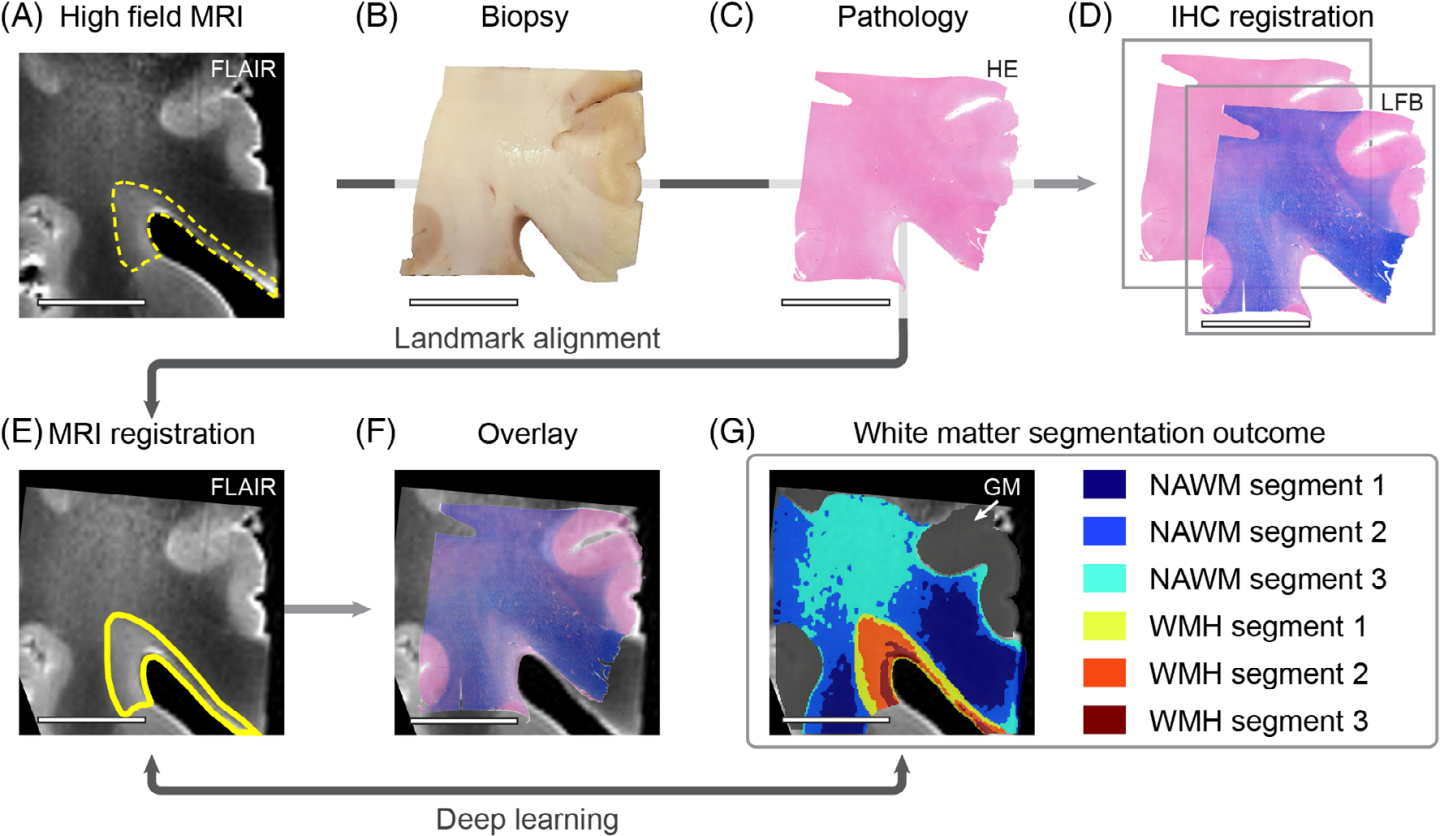
Study workflow overview. Image of corresponding high‐field MRI fluid‐attenuated inversion recovery (FLAIR) axial slab of the region of interest (A). White matter hyperintensity (WMH) before co‐registration is outlined with a yellow dotted line. The main flowchart applied for MRI co‐registration with (immuno)histochemistry (IHC) and polarized light imaging (PLI) is illustrated with dark grey arrows. Photograph of biopsy taken in order to perform IHC and PLI (B). (C) shows IHC of haematoxylin/eosin (HE). (D) Whole slide images included in this study, such as Luxol Fast Blue (LFB) and neurofilament medium chain (not shown), were co‐registered to corresponding HE. MRI‐pathology data, including IHC and PLI, was registered based on manual landmark selection. (E) depicts the outcome of the manual registration script. (F) shows the overlay between registered FLAIR and LFB. In order to examine white matter underlying pathology beyond the WMH dichotomy, we used deep learning to segment the registration outcome (E) into smaller segments of white matter, capturing the increasing fluid‐attenuated inversion recovery signal depicted in image (G). Overlay of WMH and NAWM periventricular white matter segments and grey matter (GM) are shown in image (G). Outline of WMH segmentation outcome used for further analysis is shown in image (E) (scale bar = 1 cm).

In this study, we segmented periventricular WMH and NAWM on FLAIR MRI, followed by further separation of these into smaller segments. First we used a deep learning‐based model to segment WMH, NAWM, and grey matter (GM) (Figure 1) (code is available on GitHub). Briefly, this model used T1‐weighted and FLAIR sequences and was trained on part of the manual annotations (ground truth) made by experienced neuroanatomists (GSG, BG) [25, 26]. The model consisted of ensembled fully convolutional networks in the form of U‐Nets [27], including a final “layer” that mapped the feature vectors to four prediction regions: WMH, NAWM, GM, and background. Each U‐Net model generated a probability prediction map, which was averaged and transformed into a segmentation map encompassing all predictions. We used Albumentations, a Python library [28], to expand the pool of training images. After successful segmentation into the aforementioned regions, the KMeans clustering algorithm [23] was used to classify WMH and NAWM into smaller white matter segments (1–3) based on FLAIR signal intensity (Figure 1).

### 2.3 (Immuno)histochemistry

After scanning, the left hemispheres were cut in tissue blocks of approximately 2 × 2 × 0.5 cm containing periventricular white matter. Tissue blocks (Figure 1) were embedded in paraffin and sectioned at 4 μm thickness. These sections were stained following standard (immuno)histological protocols for haematoxylin/eosin (HE), LFB, and phosphorylated neurofilament medium chain (NfM). LFB is a staining for myelin, which was used to examine myelin pallor (indirect measure for degree of myelin), whereas phosphorylated NfM, reflects axonal damage [29] and can be used to investigate axonal integrity (mouse, ncl‐nf160; Novocastra, Newcastle upon Tyne, UK; 1:400, RRID:AB_563911). Sections were stained for NfM using a fully automated immunostainer (Lab Vision Autostainer 360; Thermo Fisher Scientific, Waltham, MA, USA) and the EnVision FLEX visualization system (K8000; Agilent, Santa Clara, CA, USA; RRID:AB_2890017), according to the manufacturer's instructions. Briefly, sections were first deparaffinized in xylene, rinsed through graded ethanol series, and ultimately in demi water. Sections were rinsed in EnVision FLEX Wash Buffer (K800721‐2; Agilent, Santa Clara, CA, USA), followed by 5 min in Peroxidase‐Blocking Reagent, and a 5‐min rinse in EnVision FLEX Wash Buffer. Sections were incubated with the primary antibody for 60 min and thereafter incubated for 15 min with FLEX+ mouse (LINKER) (K802121; Agilent, Santa Clara, CA, USA). After another 10‐min rinse in EnVision FLEX Wash Buffer, sections were incubated with EnVision FLEX HRP Solution (Agilent, Santa Clara, CA, USA) for 30 min, then another 10‐min rinse in EnVision FLEX Wash Buffer. Sections were incubated with a mixture of EnVision FLEX 3,3′‐diaminobenzidine (DAB) + and Substrate Solution (Agilent, Santa Clara, CA, USA) for 10 min and rinsed in tap water for 10 min. Sections stained for NfM were counterstained using haematoxylin before dehydration in ethanol and xylene, and cover slipping.

#### 2.3.1 Quantification of degree of myelin and axonal integrity

Sections stained for myelin and axons were digitized, exported, and registered to corresponding HE sections as described elsewhere [25]. After this multimodal registration, LFB and NfM stained sections were imported and processed in ImageJ (version 1.54f, National Institute of Health, Bethesda, Maryland, United States), making use of the color deconvolution tool [30]. (Immuno)histochemistry sections were analyzed by using an intensity threshold to isolate the target staining from the background. To account for varying staining intensities across individuals, the intensity threshold was determined for each staining by examining mean intensity values of positive stained control regions. Threshold settings based on the overall mean intensity were checked on an individual basis.

### 2.4 Polarized light imaging—Acquisition and quantification

For PLI, the tissue blocks were cut in 100‐μm thick sections with a sliding freezing microtome (Microm HM 440E Microtome, Walldorf, Germany), mounted on uncoated glass slides, and cover‐slipped using the mounting medium polyvinylpyrrolidone. In‐depth PLI processing and setup have been described elsewhere [11, 17, 31]. Briefly, PLI intensity values of transmitted light directly correlate to myelin density (retardance), where lower retardance values correspond to a lower myelin density, and to white matter fiber orientation dispersal (dispersion) [17], which can be used as an indirect measure of microstructural integrity. Mean gray values (intensity) were automatically quantified using ImageJ (version 1.54f, National Institute of Health, Bethesda, MD, United States).

### 2.5 MRI‐pathology co‐registration

The MRI data (T1‐weighted, FLAIR) were compared to the HE reference section to select the corresponding 2D MRI. MRI‐(immuno)histochemistry data was co‐registered based on manual landmark selection using a custom written MATLAB script (MATLAB R2020a; MathWorks Inc., Natick, MA, USA). Briefly, MRI data were extracted from previously selected 2D axial slices. The script focused on the periventricular region, where at least 10 landmarks were selected on the MRI and HE sections. After, the MRI 2D images were warped [32] and cropped based on the reference section. 2D MRI warping was restricted to affine transformation (Figure 1). This process was repeated for PLI data to register an MRI to PLI.

In order to directly correlate MRI data with co‐registered (immuno)histochemistry and PLI at the voxel‐wise level, all co‐registered neuropathology data images were divided into 0.16 mm^2^ pixels, and the average intensity value within each pixel was calculated. For (immuno)histochemistry, intensity measurements were used to examine the degree of myelin and axonal integrity in LFB and NfM, respectively. PLI retardance map intensity values reflect myelin density, while PLI dispersion map intensity values can be used as a measure of microstructural integrity. Pixels with less than 50% of positive tissue were excluded from analysis. Finally, the average intensity value within the included pixels was used to generate heatmaps [see Figure S1].

### 2.6 Statistics

Means and standard deviation (SD) were calculated for all continuous variables, as well as frequencies and percentages for categorical variables. Relationships between categorical variables were explored using the Chi‐square test (χ^2^). We used multivariate analysis of variance (ANOVA) for group comparisons of continuous demographic variables. ANOVA, controlled for age and fixation‐(immuno)histochemistry/PLI, was used to compare myelin markers (LFB, PLI) and axonal integrity (NfM) between the brains of individuals with hypertension and controls in both WMH and NAWM, including segments. LFB and NfM parameters were corrected for age. Mean intensity values of MRI‐based segments were tested between groups and throughout white matter segments—gradual from low to high intensity—using ANOVA. All ANOVA were performed with Bonferroni correction for multiple testing. Voxel‐wise associations between MRI‐based signal and pathology throughout WMH and NAWM segments were examined using a Pearson correlation.

Statistical analysis was performed using IBM SPSS statistics 29 (IBM Corporation, Armonk, NY, USA). Alpha (2‐tailed) was set at 0.05 for descriptive analysis and ANOVA, and at 0.01 for correlations. For each pathological variable (LFB, NfM, PLI myelin density, and microstructural integrity), violin plots, including box plots, and scatter plots were generated using R software (version 4.1.3).

## 3 RESULTS

The brains of individuals with hypertension had a greater WMH burden than controls (*p* = 0.02; Table 1).

### 3.1 Degree of myelin and axonal integrity in periventricular WMH and NAWM

**FIGURE 2:**
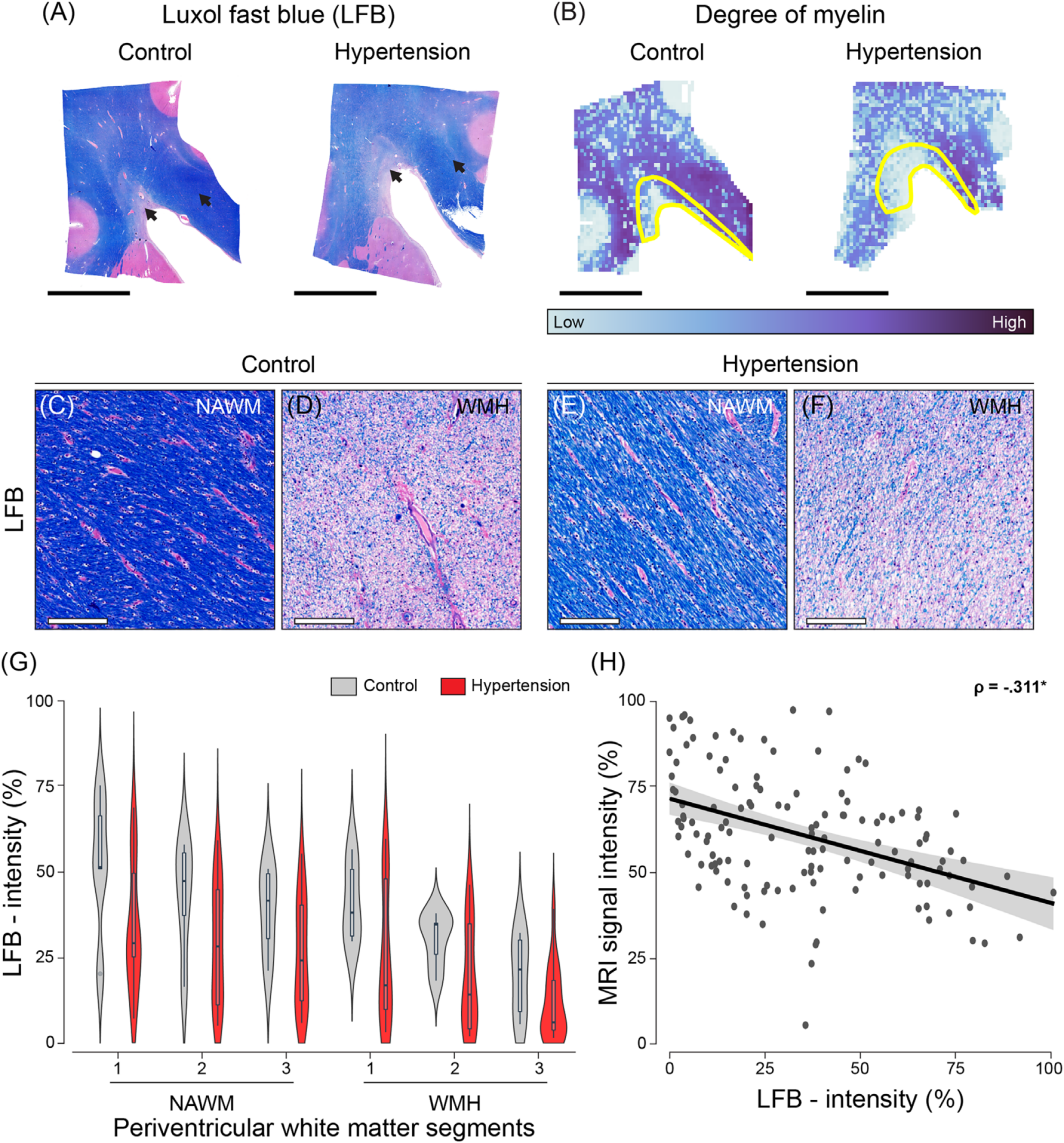
Histopathological characterization of degree of myelin in individuals with hypertension and controls throughout periventricular white matter. Representative whole slide images of Luxol Fast Blue (LFB) of the brains of normotensive individuals and individuals with hypertension (A) and their corresponding intensity quantification as heatmaps (B). The black arrows (A) indicate regions of interest visible in (C–F). The yellow outlines in B depict white matter hyperintensities (WMH). Images c and e correspond to normal‐appearing white matter (NAWM), while D and F showcase WMH. Examination of degree of myelin showed lower LFB intensity in individuals with hypertension (C, D) compared to age‐matched controls (E, F) (*p* < 0.001). LFB intensity was lower in WMH (D, F) compared to NAWM (C, E) (*p* < 0.001). Plot of LFB intensity percentage in individuals with hypertension (red) and age‐matched controls (grey) across periventricular WMH and NAWM segments shown as violin plots and respective box plots (G). For both WMH and NAWM, the periventricular white matter segment 3, which is the segment with the highest intensity visible on FLAIR, showed lower LFB intensity compared to segment 1 (*p* = 0.002). For all individuals, voxel‐wise MRI signal intensity negatively correlated with LFB intensity in both NAWM and WMH (H) (*p* < 0.001). Data points in H are shown in dark grey, while the 95% confidence interval (CI) is shown in light grey (black scale bar = 1 cm; white scale bar = 200 μm).

Degree of myelin (Figure 2) assessed through LFB intensity was lower in WMH and to a lesser extent in NAWM in individuals with hypertension compared to age‐matched controls (*p* < 0.001; Table 2). Throughout MRI‐based periventricular WMH and NAWM segments, for all individuals, the segment with the highest FLAIR signal intensity (3) contained less myelin compared to the segment exhibiting the lowest FLAIR signal intensity (1) (3 vs 1: *p* = 0.002 [see Table S1]). In particular, within WMH, the degree of myelin was 53.6% lower, while within NAWM, it was remarkably 23.9% lower.

**TABLE 2:** (Immuno)histopathology and polarized light imaging measures by groups and periventricular WMH.

	Controls		Individuals with hypertension		*p* value^a^	
	NAWM Mean ± SD	WMH Mean ± SD	NAWM Mean ± SD	WMH Mean ± SD	Groups (hypertension vs. control)	ROIs (WMH vs NAWM)
Degree of myelin [LFB]						
Intensity (%)	44.8 ± 17.0	30.5 ± 13.5	29.8 ± 17.3	18.7 ± 16.5	0.001***	0.001***
Axonal integrity [NfM]						
Intensity (%)	21.0 ± 21.6	14.1 ± 11.3	49.7 ± 25.0	35.3 ± 27.5	0.001***	0.011*
Polarized light imaging [PLI]						
Myelin density (%)	20.3 ± 10.1	17.8 ± 7.4	22.0 ± 6.5	18.9 ± 7.9	0.317	0.043*
Microstructural integrity (%)	90.5 ± 8.0	90.1 ± 5.3	90.5 ± 4.0	89.6 ± 7.2	0.795	0.586

*Note*: *p < 0.05; **p < 0.01; ***p < 0.001.

Abbreviations: LFB, Luxol Fast Blue; NAWM, normal‐appearing white matter; NfM, neurofilament medium chain; PLI, polarized light imaging; ROIs, regions of interest; SD, standard deviation; WMH, white matter hyperintensity.

^a^ *p* values represent values after Bonferroni correction.

**FIGURE 3:**
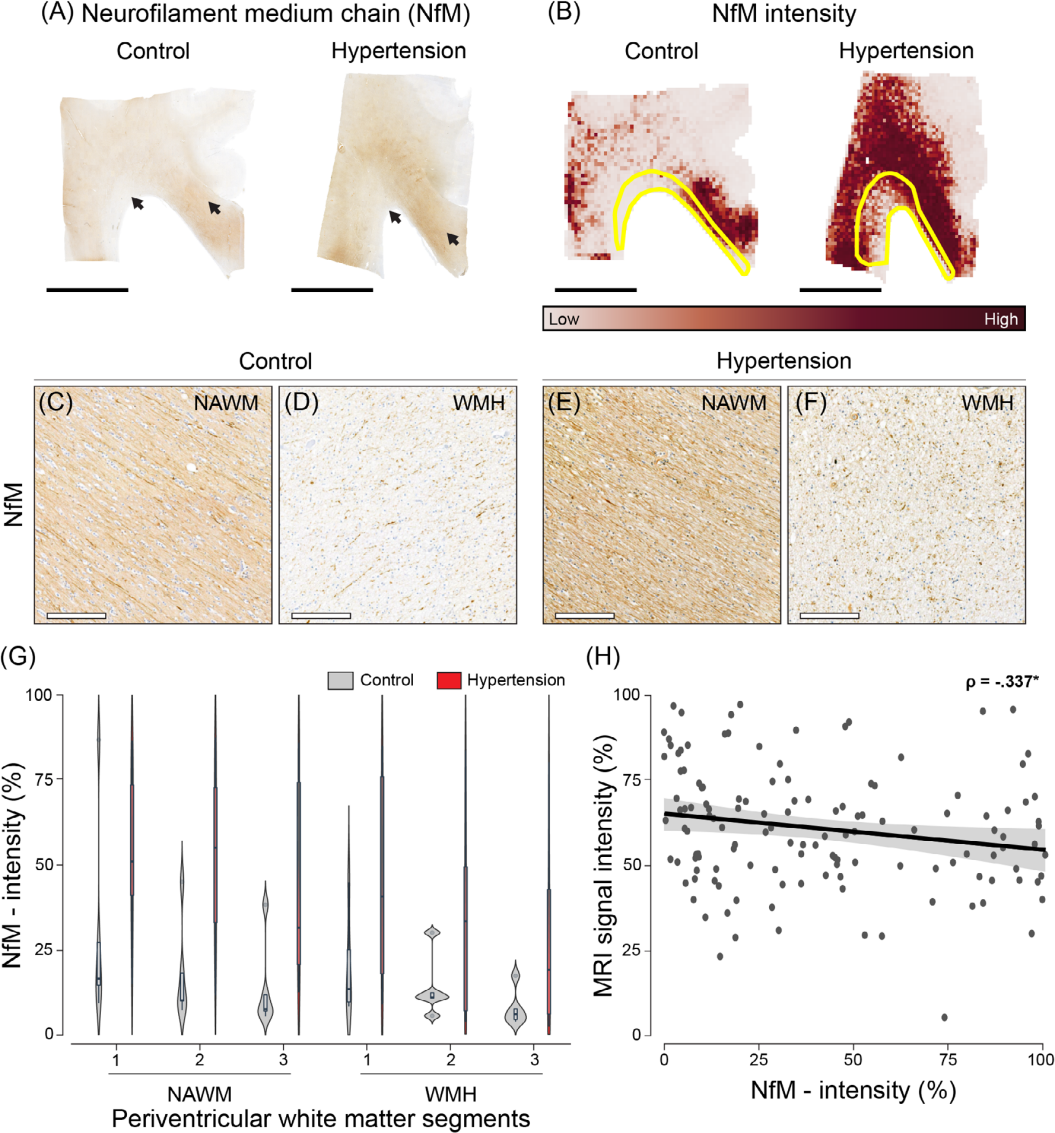
Immunohistopathological characterization of axonal integrity of individuals with hypertension and controls throughout periventricular white matter. Representative whole slide images of phosphorylated Neurofilament medium chain (NfM) of brains of normotensive individuals and individuals with hypertension (A) and their corresponding intensity quantification as heatmaps (B). The black arrows (A) indicate regions of interest visible in C–F. The yellow outlines in image B depict white matter hyperintensities (WMH). Images C and E correspond to normal‐appearing white matter (NAWM), while D and F showcase WMH. We found higher NfM intensity in brains of individuals with hypertension (C, D) compared to age‐matched controls (E, F) (*p* < 0.001). For all individuals, NfM intensity was lower in WMH (D, F) compared to NAWM (C, E) (*p* = 0.011). Plot of NfM intensity percentage in the brains of individuals with hypertension (red) and age‐matched controls (grey) across periventricular WMH and NAWM segments shown as violin plots and respective box plots (G). For both WMH and NAWM, the periventricular white matter segment 3, which is the segment with the highest intensity visible on FLAIR for both NAWM and WMH, showed lower NfM intensity compared to segment 1 (*p* = 0.011). For all individuals, voxel‐wise MRI signal intensity negatively correlated with NfM intensity in both NAWM and WMH (H) (*p* < 0.001). Data points in H are shown in dark grey, while the 95% confidence interval (CI) is shown in light grey (black scale bar = 1 cm; white scale bar = 200 μm).

NfM intensity (Figure 3), which reflects axonal damage [29], was 2.4 fold higher in both periventricular WMH and NAWM of individuals with hypertension than controls (*p* < 0.001; Table 2). However, for all individuals, NfM intensity was lower in WMH than NAWM (*p* = 0.011; Table 2). Similarly, throughout both periventricular WMH and NAWM MRI‐based segments, the segment with the highest FLAIR signal intensity showed lower NfM intensity compared to the segment with the lowest FLAIR signal intensity (1) (3 vs 1: *p* = 0.011 [see Table S1]). See Figure S2 for (immuno)histochemistry of representative whole slide images of normal white matter of control individuals stained for LFB and NfM.

### 3.2 PLI assessment of myelin density and microstructure in periventricular WMH and NAWM

**FIGURE 4:**
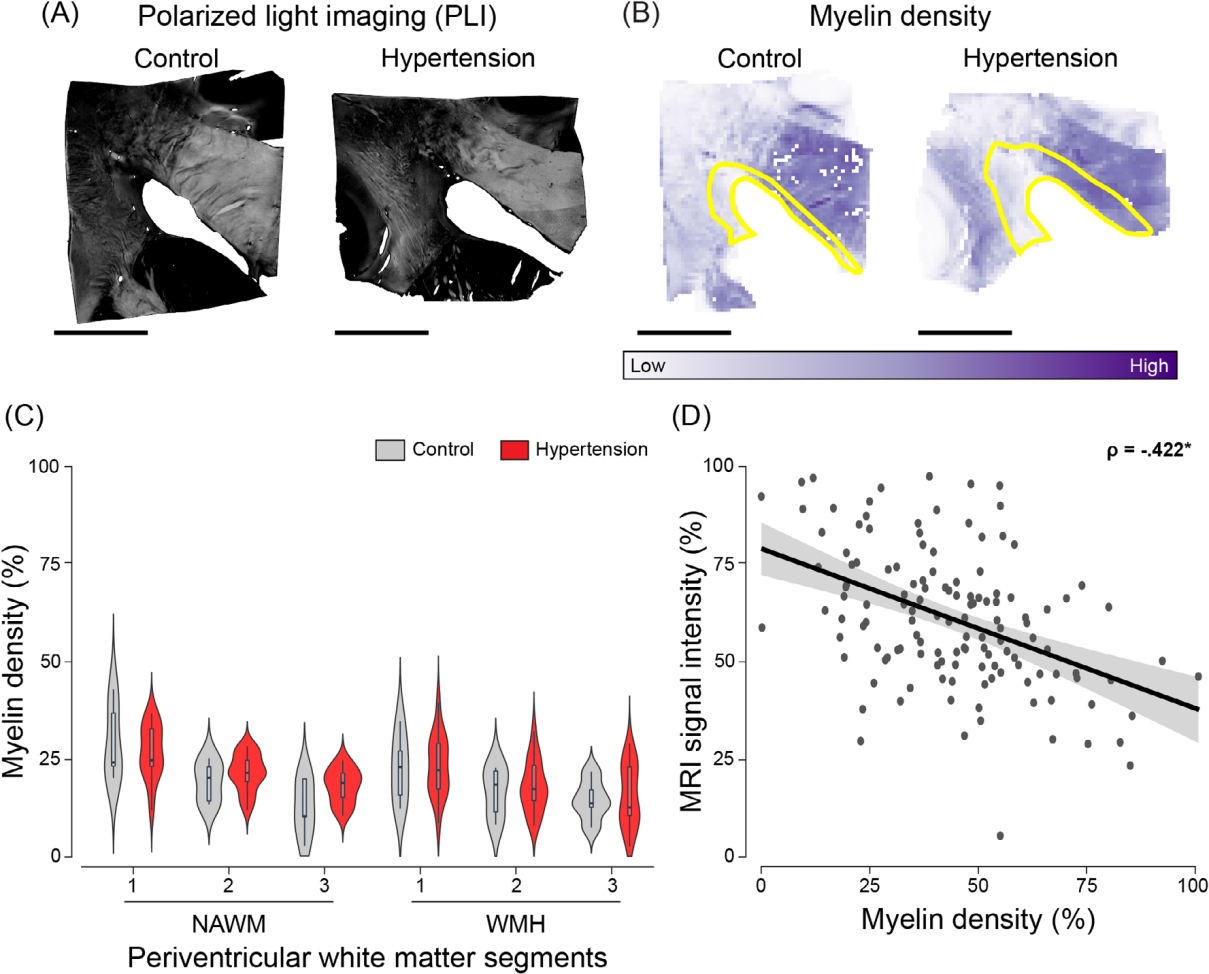
Polarized light imaging myelin density in individuals with hypertension and controls throughout periventricular white matter. Representative images of myelin density as seen through polarized light imaging (PLI) of the brains of normotensive individuals and individuals with hypertension (A) and their corresponding quantification as heatmaps (B). The yellow outlines in image B depict white matter hyperintensities (WMH). Examination of myelin density showed lower myelin density in WMH compared to normal‐appearing white matter (NAWM) in both individuals with hypertension and age‐matched controls (*p* = 0.043). Plot of myelin density percentage in brains of individuals with hypertension (red) and age‐matched controls (grey) across periventricular WMH and NAWM segments shown as violin plots and respective box plots (C). For both WMH and NAWM, we found that the white matter segment 1, which is the segment with the lowest intensity visible on FLAIR, had higher myelin density than segment 2 (*p* < 0.001) and 3 (*p* < 0.001). For all individuals, voxel‐wise MRI signal intensity negatively correlated with myelin density in both NAWM and WMH (D) (*p* < 0.001). Data points in D are shown in dark grey, while the 95% confidence interval (CI) is shown in light grey (black scale bar = 1 cm).

We did not observe myelin density differences between individuals with hypertension and age‐matched controls in the periventricular WMH and NAWM (Figure 4; Table 2). We found 13.2% lower myelin density in the periventricular WMH compared to NAWM for both groups (*p* = 0.043; Table 2). Assessment of myelin density throughout periventricular MRI‐based segments showed that for both WMH and NAWM, the white matter segment with the lowest FLAIR signal intensity (1) had higher myelin density than the other segments (1 vs. 2: *p* < 0.001; 1 vs. 3: *p* < 0.001 [see Table S1]).

**FIGURE 5:**
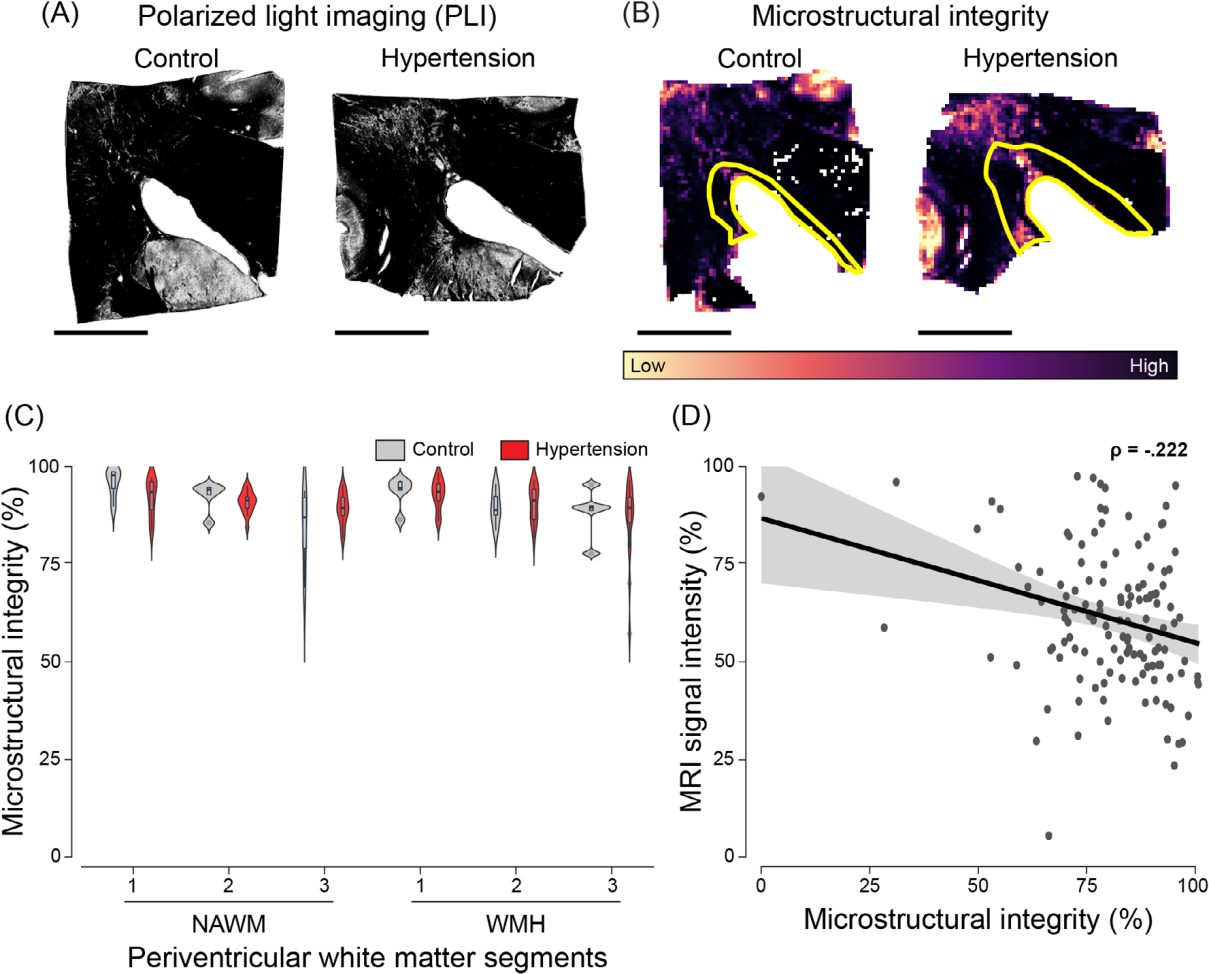
Polarized light imaging microstructural integrity in individuals with hypertension and controls throughout periventricular white matter. Representative images of myelin microstructure as seen through polarized light imaging (PLI) of the brains of normotensive individuals and individuals with hypertension (A) and their corresponding quantification as heatmaps (B). The yellow outlines in image B depict white matter hyperintensities (WMH). We found no differences in microstructural integrity between groups or WMH. Plot of myelin microstructure integrity percentage in brains of individuals with hypertension (red) and age‐matched controls (grey) across periventricular WMH and NAWM segments shown as violin plots and respective box plots (C). For both WMH and NAWM, we found that the white matter segment 3, which is the segment with the highest intensity visible on FLAIR, had lower microstructural integrity than segment 1 (*p* = 0.002). D Plot illustrating MRI signal intensity (y‐axis) and microstructural integrity (x‐axis). Data points in D are shown in dark grey, while the 95% confidence interval (CI) is shown in light grey (black scale bar = 1 cm).

We found no differences in myelin microstructural integrity assessed with PLI between individuals with hypertension and age‐matched control individuals or between periventricular WMH and NAWM (Figure 5; Table 2). However, when assessing myelin microstructure throughout MRI‐based segments, we found that for both WMH and NAWM, the segment with the highest intensity visible on FLAIR showed 6% more compromised myelin microstructure compared to the other segments (3 vs. 2: *p* = 0.023; 3 vs. 1: *p* < 0.001 [see Table S1]).

### 3.3 Voxel‐wise myelin loss and altered axonal integrity throughout periventricular WMH and NAWM

To examine the underlying pathology of (increased) MRI signal intensity, we correlated voxel‐wise FLAIR signal to (immuno)histochemistry and PLI parameters throughout deep learning‐based periventricular WMH and NAWM segments. Voxel‐wise MRI signal negatively correlated with both degree of myelin (LFB: ρ = −0.311, *p* < 0.001, Figure 2H; PLI: ρ = −0.422, *p* < 0.001, Figure 4D) and NfM immunolabeling (NfM: ρ = −0.337, *p* < 0.001, Figure 3H). Altogether, these findings suggest that high field MRI is sensitive to subtle changes in myelin density and axonal damage not only in WMH but also within NAWM.

## 4 DISCUSSION

We found two‐fold larger axonal damage in periventricular WMH and NAWM in the brains of individuals with hypertension compared to controls without hypertension. Notably, we demonstrated that myelin loss, impaired axonal integrity, and white matter microstructural damage are already present in NAWM. In addition, higher voxel‐wise FLAIR signal intensity in periventricular WMH and NAWM was associated with lower myelin density and axonal integrity. This suggests that MRI can indirectly assess white matter degeneration, extending beyond current definitions of WMH.

In line with prior evidence [7], we observed a lower degree of myelin (assessed with LFB staining) and myelin density (measured with PLI) in periventricular WMH compared to NAWM. Notably, our findings highlight interesting differences between LFB, histology's gold standard, and PLI when assessing myelin differences across individuals with and without hypertension. On the one hand, LFB revealed lower degree of myelin in individuals with hypertension compared to controls, whereas PLI showed no differences in myelin density between groups. This discrepancy likely stems from the inherent differences between these techniques. LFB became the gold standard post‐mortem technique to study the degree of myelin [33]. However, LFB relies on dyes binding to various components of myelin; thus, the observation of myelin pallor can have several etiologies, such as the presence of edema [13], which is common in hypertension. Additionally, LFB staining is not only related to myelin density but also to the lipid composition of myelin [34]. Therefore, an explanation for the decreased LFB staining but unchanged PLI found in our study may be caused by a possible reduction of certain lipid components within the myelin sheath. Other studies may also come across these contrasting findings when using LFB staining and immunohistochemistry for myelin protein [35]. PLI relies on the intrinsic birefringent properties of the myelin sheaths surrounding the axons coupled with independent component analysis (theory is extensively described elsewhere [14, 15]), offering a more objective measure for myelin density. Nonetheless, its application to study white matter damage is relatively new [11, 16], and its results should therefore be interpreted with care. These results suggest that actual myelin content in periventricular NAWM may remain relatively stable in individuals with hypertension compared to controls, which cannot be concluded with LFB. This underlines the importance of considering technical limitations when evaluating myelin density by proxy in the context of hypertension since immunohistochemistry and/or PLI might overcome the limitations of LFB. Hence we also used PLI, which has been used before to assess myelin microstructure and validate advanced diffusion MRI metrics [17]. Briefly, PLI's measures for microstructural integrity correlate with orientation dispersion index [17], which quantifies the spread of fibers within the intracellular compartment. The orientation dispersion index offers a more specific estimate of microstructural integrity than mean diffusivity [36]. Thus, microstructural integrity (measured with PLI) in the WMH–NAWM border zone can provide unique insights to the benefit of advanced diffusion MRI metrics in SVD clinical research to understand white matter microstructural damage in these border zones. Remarkably, we found lower myelin density and microstructural integrity throughout automatically segmented periventricular sections in the WMH and NAWM border zone, complementing previous diffusion MRI evidence suggesting the presence of structural white matter alterations within NAWM [37]. Altogether, our novel PLI and LFB findings highlight the need for more specific in vivo myelin imaging such as quantitative MRI and advanced diffusion MRI to accurately unravel the contribution of structural changes and myelin loss in the pathogenesis of SVD since both processes seem to be present already within NAWM. Fixel‐based advanced diffusion MRI single‐shell 3‐tissue constrained spherical deconvolution (SS3T‐CSD) may be such an approach to further characterize microstructural composition in vivo [19, 20].

Furthermore, our study demonstrated that phosphorylated NfM staining intensity, used to measure axonal integrity, was over two‐fold higher in WMH and NAWM of individuals with hypertension compared to controls. To the best of our knowledge, no studies have previously examined phosphorylated NfM in the periventricular white matter of individuals with hypertension. However, in other neurodegenerative diseases such as Alzheimer's disease [38, 39], multiple sclerosis [40]. and amyotrophic lateral sclerosis [41, 42], increased accumulation of phosphorylated NfM is well established. Neurofilaments are essential for structural stability and synaptic function, and the accumulation of phosphorylated NfM may have several reasons. For example, stressful conditions, including ischemic damage and inflammation, can increase the phosphorylation of NfM through hyperactivation of the cyclin‐dependent kinase 5/p25 complex [43] leading to axonal damage of nerve fibers. Additionally, accumulation of phosphorylated NfM may reflect disturbances in axonal transport as well as ongoing Wallerian degeneration [44, 45]. Thus, increased accumulation of phosphorylated NfM in axons not necessarily shows initial axonal damage but could also be a secondary consequence of axonal damage. While we cannot infer causality, in our recent post‐mortem study we observed that neuro(vascular) inflammation was two‐fold higher in individuals with hypertension [25], which may worsen hypertension‐induced endothelial damage and atherosclerosis [46] leading to ischemia and neuronal damage. These exacerbated stressful conditions in individuals with hypertension could explain the larger NfM intensity reflecting axonal damage observed in this study. Eventually, this can lead to disrupted axonal stability and function, ultimately causing axonal and myelin loss [45]. In fact, we found lower NfM intensity within WMH of both groups, which may reflect that in WMH there is a large axonal loss, unlike the increased NFM intensity reflecting ongoing axonal damage of nerve fibers that are still present. Therefore, NfM accumulation may precede the conversion into WMH. Consequently, future studies are needed to elucidate the association of early‐stage processes such as blood–brain barrier leakage and neuro(vascular) inflammation [47] with increased levels of neurofilaments, particularly in individuals with hypertension.

This study has several important clinical implications. First, our findings strengthen the relevance of neurofilament not only as a biomarker for active SVD [48], but also its sensitivity to early axonal damage. In this study, we showed that hypertension was associated with elevated NfM in NAWM and to a lesser extent in WMH. This suggests that axonal damage might precede myelin and axonal loss, and consequently, WMH conversion. Second, our findings on voxel‐wise myelin loss and altered axonal integrity beyond the boundaries of WMH into the NAWM highlight that the dichotomous examination of WMH may only capture the “tip of the iceberg.” These findings, combined with the knowledge that higher voxel‐wise FLAIR intensity values in NAWM correlate with a greater likelihood of WMH conversion [37], imply an added benefit of using highly reproducible imaging segmentation methods such as KMeans [23] in clinical studies. Likewise, in this study we showed for the first time that further segmentation within periventricular WMH and NAWM reflects different degrees of white matter microstructural damage. Notably, the severity of white matter microstructural damage gradually progressed throughout the periventricular white matter until the center of WMH. Therefore, further segmentation methods may recapitulate different degrees of white matter microstructural damage, including earlier stages, in both NAWM and WMH. Characterization of the continuum of white matter pathology may lead to improved patient care since it could support unraveling WMH heterogeneity as well as individuals' risk to macrostructural white matter damage.

To the best of our knowledge, this is the first post‐mortem study examining white matter microstructure throughout periventricular WMH and NAWM segments using deep learning segmentation based on FLAIR signal. A limiting factor of post‐mortem studies is their often small sample size, hindering deep learning model training. However, by using deep learning methods that can generate more training images [28], we were able to achieve robust model training for WMH segmentation even with our limited sample size. While this novel approach allowed us to uniquely characterize subvisible white matter microstructural abnormalities, we acknowledge that the observed difference in compromised myelin microstructure within WMH may appear modest despite being statistically significant. Nonetheless, we implemented Bonferroni correction to mitigate the risk of Type I error. Moreover, although we assessed axonal integrity using phosphorylated NfM immunolabeling, future studies are needed to elucidate the reactivity of each neurofilament subunit to processes such as neuroinflammation. As the neurofilament light chain is increasingly recognized as a predictor for cognitive decline [49, 50], elucidating the extent of axonal damage where these lesions occur is critical to completely understand the significance of neurofilaments as biomarkers in the context of SVD. Finally, although high‐field imaging‐pathology evidence of myelin and axonal loss in the context of SVD is very limited, with only one study examining this specific aspect to date [51], our findings extend on prior research examining post‐mortem (neuro)vascular inflammation [25]. Over recent years, circulating neurofilament light chain and glial fibrillary acidic protein have been independently and jointly associated with dementia risk [52]. The direct correlation between these markers in SVD is still missing. Application of voxel‐wise pathological characterization might be considered in future research to directly characterize the association between axonal damage and inflammation. Furthermore, future post‐mortem studies including markers for vascular alterations such as immune cells (infiltration) are needed to further elucidate high‐field MRI sensitivity to subtle early‐stage neuropathological changes in SVD. Future studies should also consider staining for amyloid beta to better elucidate the contribution of small vessel disease from cerebral amyloid angiopathy.

## 5 CONCLUSIONS

In conclusion, our findings strongly imply that elevated axonal damage in SVD may play a role in the conversion into WMH. Furthermore, given that both microstructural alterations and myelin and axonal loss might already occur in NAWM, the integration of more specific in vivo myelin imaging such as quantitative MRI and advanced diffusion MRI metrics is needed to further elucidate the contribution of these processes to the pathogenesis of SVD and ultimately to cognitive decline. Moreover, as machine learning algorithms become more accessible, similar segmentation approaches to study early, subvisible white matter microstructural changes could be applied to in vivo data, complementing the output of standard sequences, for example, FLAIR, without extending the imaging protocols.

## AUTHOR CONTRIBUTIONS

AJK, FEdL, MW, JAHRC, and GSG conceptualized and designed the study. AJK, MW, GSG, EJ, RV, and BG contributed to the material preparation and data collection. GL, ML, and GSG designed and implemented the computer code. GSG analyzed the data with support from AJK, FEdL, MW, JAHRC, and BK. GSG, AJK, FEdL, MW, and JAHRC drafted the manuscript, and all the authors reviewed it and provided feedback.

## FUNDING INFORMATION

MW is supported by Alzheimer Nederland (grant No. WE.15–2021‐10).

## CONFLICT OF INTEREST STATEMENT

No actual or potential conflicts of interests apply.

## ETHICS STATEMENT

All individuals signed informed consent to use their medical records for research purposes, autopsy, and use of tissue. The study was approved by the Medical Ethics Review Committee region Arnhem‐Nijmegen (Commissie Mensgebonden Onderzoek (CMO) region Arnhem‐Nijmegen file No. 2017–3941).

## Supporting information


**Figure S1.** (Immuno)histochemistry and polarized light imaging analysis workflow. Tissue blocks of approximately 2 × 2 × 0.5 cm were taken for (immuno)histochemistry and polarized light imaging (PLI). Image a depicts a section stained for Luxol Fast Blue (LFB). From left to right, images b–c show their corresponding color deconvolution (b) and quantification outcome as MATLAB generated heatmaps (c). Image d depicts a section stained for phosphorylated neurofilament medium chain (NfM). Image e shows their corresponding color deconvolution and f the quantification outcome as heatmap. Additionally, we performed polarized light imaging, resulting in a fiber optic dispersion map (g), myelin density [retardance] (h), and myelin microstructure [dispersion] (j). Images i and k depict the quantification outcome of myelin density and microstructural integrity, respectively, as heatmaps (scale bar = 1 cm) (TIFF 5872 kb).

**Table S1.** (Immuno)histopathology and polarized light imaging measures across periventricular WMH and NAWM, and their respective segments (PDF 130 kb).

**Figure S2.** (Immuno)histochemistry of control individuals, including periventricular white matter and normal white matter. Representative whole slide images of Luxol Fast Blue (LFB) of periventricular and normal white matter of individuals without hypertension (A). Representative whole slide images of phosphorylated neurofilament medium chain (NfM) of periventricular and normal white matter of individuals without hypertension (B) (scale bar = 1 cm) (TIFF 2101 kb).

## Data Availability

The datasets generated and/or analyzed during the current study are available upon reasonable request to the corresponding author.
