# Supplementary figures and images for: Deep learning‐based segmentation in MRI‐(immuno)histological examination of myelin and axonal damage in normal‐appearing white matter and white matter hyperintensities
Source: Brain Pathol. 2024 Aug 23;35(2):e13301. doi: 10.1111/bpa.13301 (PMC11835442; doi:10.1111/bpa.13301)

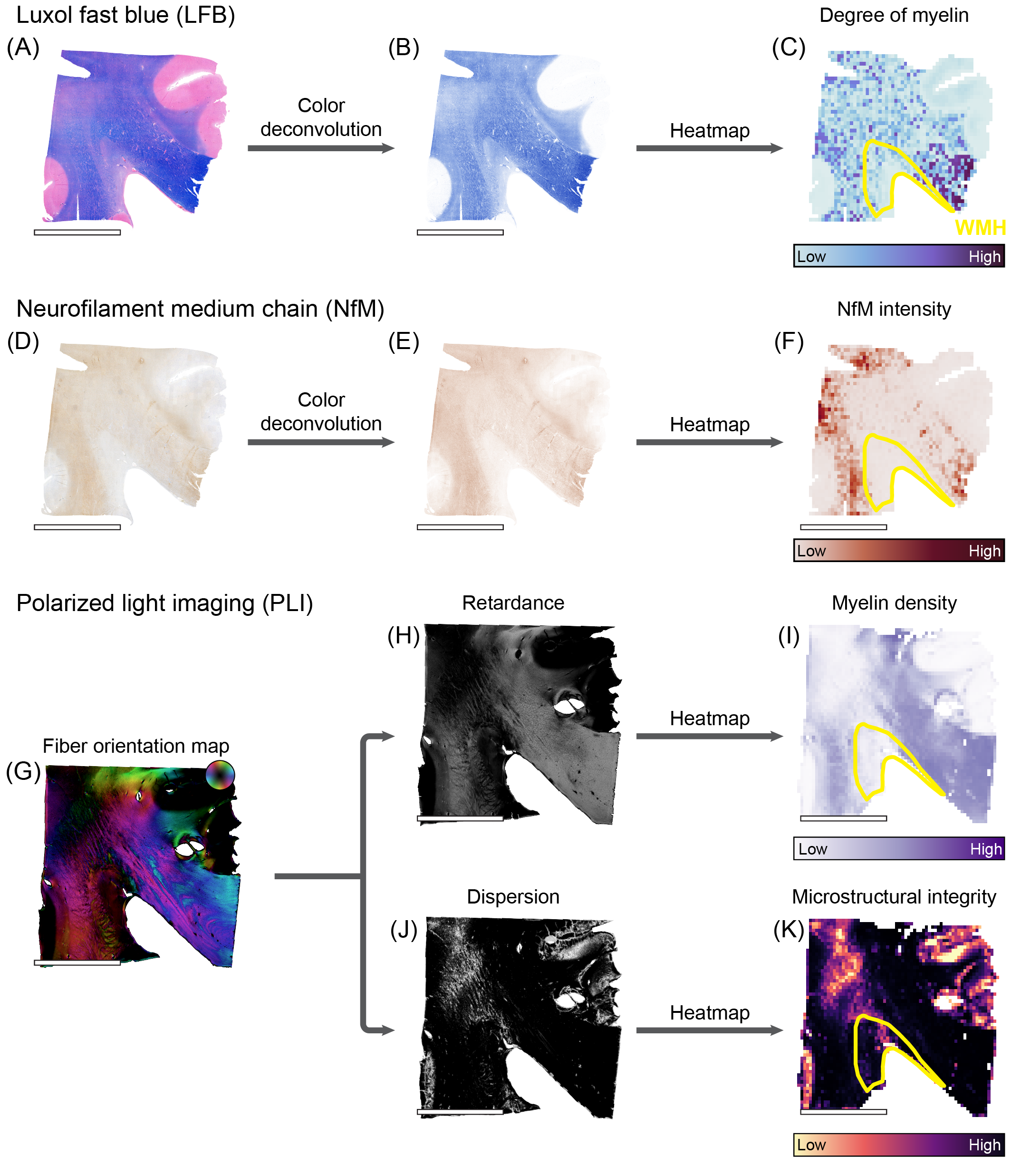

Supplement: Supplementary file 1 — Figure S1. (Immuno)histochemistry and polarized light imaging analysis workflow. Tissue blocks of approximately 2 × 2 × 0.5 cm were taken for (immuno)histochemistry and polarized light imaging (PLI). Image a depicts a section stained for Luxol Fast Blue (LFB). From left to right, images b–c show their corresponding color deconvolution (b) and quantification outcome as MATLAB generated heatmaps (c). Image d depicts a section stained for phosphorylated neurofilament medium chain (NfM). Image e shows their corresponding color deconvolution and f the quantification outcome as heatmap. Additionally, we performed polarized light imaging, resulting in a fiber optic dispersion map (g), myelin density [retardance] (h), and myelin microstructure [dispersion] (j). Images i and k depict the quantification outcome of myelin density and microstructural integrity, respectively, as heatmaps (scale bar = 1 cm) (TIFF 5872 kb). [file BPA-35-e13301-s003.tif]

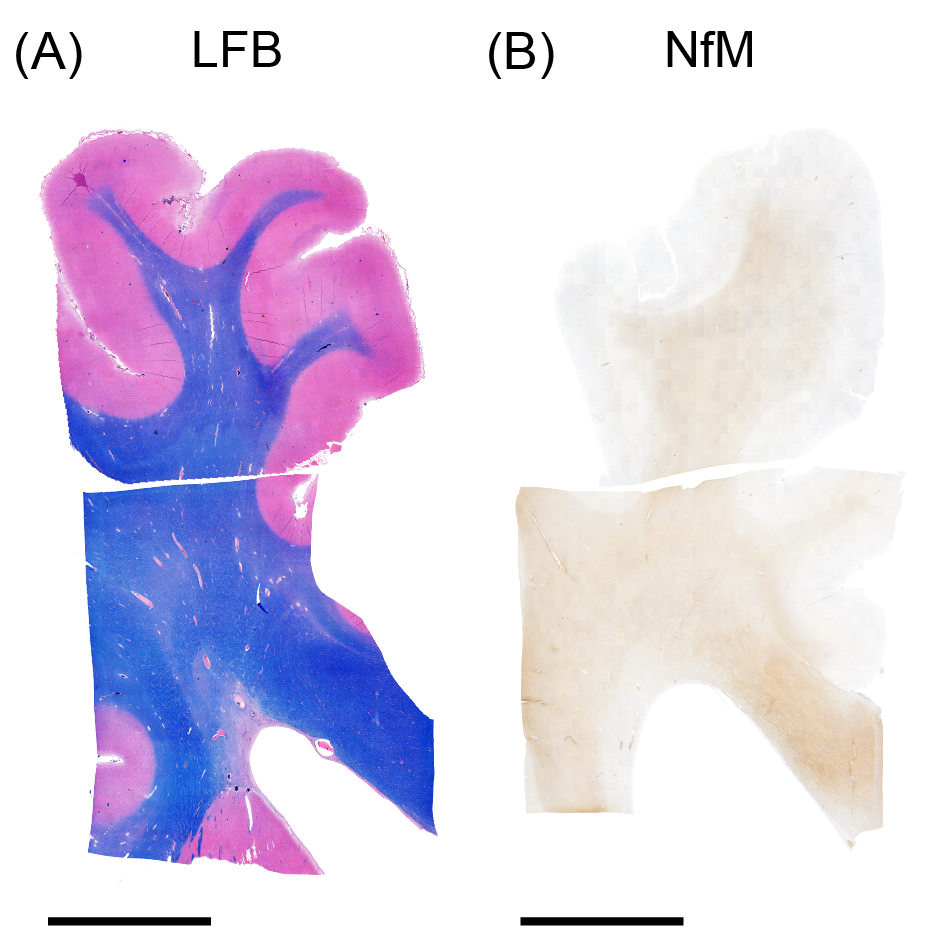

Supplement: Supplementary file 3 — Figure S2. (Immuno)histochemistry of control individuals, including periventricular white matter and normal white matter. Representative whole slide images of Luxol Fast Blue (LFB) of periventricular and normal white matter of individuals without hypertension (A). Representative whole slide images of phosphorylated neurofilament medium chain (NfM) of periventricular and normal white matter of individuals without hypertension (B) (scale bar = 1 cm) (TIFF 2101 kb). [file BPA-35-e13301-s002.tif]
