# Supplementary material for: Deep learning‐based segmentation in MRI‐(immuno)histological examination of myelin and axonal damage in normal‐appearing white matter and white matter hyperintensities
Source: Brain Pathol. 2024 Aug 23;35(2):e13301. doi: 10.1111/bpa.13301 (PMC11835442; doi:10.1111/bpa.13301)
Supplement: Supplementary file 2 — Table S1. (Immuno)histopathology and polarized light imaging measures across periventricular WMH and NAWM, and their respective segments (PDF 130 kb). [file BPA-35-e13301-s001.pdf]

Table S1 (Immuno)histopathology and polarized light imaging measures across periventricular WMH and NAWM, and their respective segments

	**NAWM**			**WMH**			***p value*** [a]		
	Periventricular white matter segments			***Periventricular white matter segments***			***Periventricular white matter segments***		
	**1** mean ± SD	**2** mean ± SD	**3** mean ± SD	**1** mean ± SD	**2** mean ± SD	**3** mean ± SD	***(1 vs 2)***	***(2 vs 3)***	***(1 vs 3)***
Degree of myelin [LFB]									
Intensity (%)	38.1 ± 20.3	32.4 ± 17.9	29.0 ± 16.0	28.9 ± 19.4	21.7 ± 14.7	13.4 ± 11.2	*p = 0.164*	*p = 0.356*	***p = 0.002 *****
Axonal integrity [NfM]									
Intensity (%)	49.5 ± 26.7	44.1 ± 26.4	36.0 ± 27.4	38.8 ± 27.9	30.5 ± 26.0	22.1 ± 23.2	*p = 0.375*	*p = 0.475*	***p = 0.011 ****
Polarized light imaging [PLI]									
Myelin density (%)	27.3 ± 7.5	20.9 ± 4.9	16.8 ± 5.5	23.0 ± 7.8	17.5 ± 6.4	15.4 ± 7.3	***p < 0.001 ******	*p = 0.117*	***p < 0.001 ******
Microstructural integrity (%)	92.7 ± 4.8	91.0 ± 3.0	87.8 ± 6.1	92.7 ± 3.8	90.0 ± 4.5	86.4 ± 9.2	*p = 0.176*	***p = 0.023 ****	***p = 0.002 *****

NAWM and WMH [1-3] correspond to periventricular white matter segments, where higher segments numbers correspond to higher intensity FLAIR values. LFB Luxol Fast Blue, MRI magnetic resonance imaging, NAWM normal-appearing white matter, NfM neurofilament medium chain, PLI polarized light imaging, SD standard deviation, WMH white matter hyperintensity. *p<0.05, **p<0.01, ***p<0.001

[a] *p* values represent values after Bonferroni correction
